# Patient perspective on remote monitoring of cardiovascular implantable electronic devices: rationale and design of the REMOTE-CIED study

**DOI:** 10.1007/s12471-014-0587-z

**Published:** 2014-08-19

**Authors:** H. Versteeg, S. S. Pedersen, M. H. Mastenbroek, W. K. Redekop, J. O. Schwab, P. Mabo, M. Meine

**Affiliations:** 1 Department of Cardiology, University Medical Center Utrecht, PO Box 85500, 3508 GA Utrecht, the Netherlands; 2 CoRPS–Center of Research on Psychology in Somatic Diseases, Department of Medical and Clinical Psychology, Tilburg University, P.O. Box 90153, 5000 LE, Tilburg, the Netherlands; 3 Department of Psychology, University of Southern Denmark, Campusvej 55, 5230 Odense M, Denmark; 4 Department of Cardiology, Odense University Hospital, Sdr. Boulevard 29,5000, Odense C, Denmark; 5 Department of Cardiology, Erasmus Medical Center, P.O. Box 2040, 3000 CA, Rotterdam, the Netherlands; 6 Institute for Medical Technology Assessment, Erasmus University Rotterdam, PO Box 1738, 3000 DR Rotterdam, the Netherlands; 7 Department of Medicine–Cardiology, University of Bonn, Seigmund-Freud-Str.25, 53127 Bonn, Germany; 8 Service de Cardiologie, Centre Hospitalier Universitaire, 16 Boulevard de Bulgarie, 35200 Rennes, France

**Keywords:** REMOTE-CIED, Cardiovascular implantable electronic devices, Remote monitoring, Patient-reported outcomes, Cost-effectiveness

## Abstract

**Background:**

Remote patient monitoring is a safe and effective alternative for the in-clinic follow-up of patients with cardiovascular implantable electronic devices (CIEDs). However, evidence on the patient perspective on remote monitoring is scarce and inconsistent.

**Objectives:**

The primary objective of the REMOTE-CIED study is to evaluate the influence of remote patient monitoring versus in-clinic follow-up on patient-reported outcomes. Secondary objectives are to: 1) identify subgroups of patients who may not be satisfied with remote monitoring; and 2) investigate the cost-effectiveness of remote monitoring.

**Methods:**

The REMOTE-CIED study is an international randomised controlled study that will include 900 consecutive heart failure patients implanted with an implantable cardioverter defibrillator (ICD) compatible with the Boston Scientific LATITUDE® Remote Patient Management system at participating centres in five European countries. Patients will be randomised to remote monitoring or in-clinic follow-up. The In-Clinic group will visit the outpatient clinic every 3–6 months, according to standard practice. The Remote Monitoring group only visits the outpatient clinic at 12 and 24 months post-implantation, other check-ups are performed remotely. Patients are asked to complete questionnaires at five time points during the 2-year follow-up.

**Conclusion:**

The REMOTE-CIED study will provide insight into the patient perspective on remote monitoring in ICD patients, which could help to support patient-centred care in the future.

## Introduction

Given the growth in the number of patients receiving a cardiovascular implantable electronic device (CIED), in particular the implantable cardioverter defibrillator (ICD) and cardiac resynchronisation therapy (CRT) devices, the burden on outpatient clinics to follow up these patients is increasing [1–3]. Remote patient monitoring offers a potential solution to this problem [4]. Remote monitoring systems contain a home transmitter that interrogates the CIED at pre-specified time points and sends the acquired data (e.g. information on battery status, lead impedances) from the patient’s home to the hospital, hereby avoiding or reducing the number of unnecessary in-clinic visits. In between these scheduled remote follow-ups, the transmitter sends information to the physician on technical (e.g., device integrity) or clinical (e.g., arrhythmias) issues, which are checked regularly [4]. Besides device parameters, some remote monitoring systems include tools to monitor the clinical status of the patient, such as fluid status, weight and blood pressure. By frequent assessment of these parameters, remote monitoring allows the early detection of heart failure worsening which might prevent hospitalisations [5].

Multiple large-scale clinical trials in ICD patients have shown that remote monitoring significantly reduces the number of in-clinic visits, without impairing patient safety [6]. Additionally, remote patient monitoring reduces the time from onset of events (e.g. arrhythmias and device malfunctions) to clinical decision-making compared with conventional in-clinic follow-ups, which may also lead to a reduction in ICD shocks [6, 7]. Most current ICDs are capable of remote monitoring and several professional societies recommend the routine use of remote monitoring in clinical practice [2, 4].

Recent surveys in the United States and Europe demonstrated that although the use of remote monitoring is growing, overall adaptation remains low and varies considerably between hospitals [8, 9]. Impediments to implementation include the absence of national guidelines and reimbursement models for remote monitoring in clinical practice [4, 6, 9–11]. Also, remote monitoring is unlikely to become the standard of care until it is conclusively demonstrated that patient outcome is favourably affected [12, 13]. Whether clinical effects of remote monitoring translate into improved patient-reported outcomes such as symptoms, quality of life and satisfaction with care, and whether patients are more satisfied with remote monitoring than in-clinic follow-up has received little attention.

To date, the few randomised controlled trials on remote monitoring in ICD patients that included patient-reported outcomes have yielded inconsistent results [14–16]. Prospective cohort studies using short ad hoc questionnaires have reported that 60–95 % of ICD patients are highly satisfied with remote monitoring and that it makes them feel safe [17–22]. However, these studies provided no information on how many patients refused to use a remote monitoring system and preferred to be checked at the outpatient clinic. This is essential, as a recent registry study from the United States showed that 24 % of the patients who received a remote monitoring system did not activate it [8].

No prospective randomised study has examined whether there is a subset of patients who might not benefit from remote monitoring in terms of patient-reported outcomes and might be more satisfied with standard clinical follow-up visits. For example, a substantial number of ICD patients report increased emotional distress, such as anxiety and depression [23]. The remote monitoring system might provide these patients with a sense of security, but on the other hand the system may act as a constant reminder of their device and underlying disease. Also, patients might miss the personal attention and reassurance from their treating physician and heart failure team [24]. A qualitative study on ICD patients who received a remote monitoring system showed that nonusers believed that in-clinic visits are psychologically advantageous and trusted the healthcare professionals over technology when it came to managing their health [25].

The international REMOTE-CIED study is the first prospective, randomised controlled study primarily designed to examine the patient perspective on remote monitoring in ICD patients, in order to enhance the patient-centredness of care in this patient group. The primary study objective is to evaluate the influence of remote patient monitoring versus conventional in-clinic follow-up on patient-reported outcomes, by means of standardised and validated questionnaires. Secondary objectives are to (1) identify subgroups of patients who might not be satisfied with remote monitoring and examine if they are distinguishable based on their demographic, clinical, or psychological profile; and (2) investigate the cost-effectiveness of remote monitoring + in-clinic follow-up as compared with in-clinic follow-up only.

## Methods

### Study design

The REMOTE-CIED study is a multicentre, prospective, randomised controlled study, with patients being recruited from five European countries (i.e., France, Germany, Spain, Switzerland and the Netherlands). The University Medical Centre Utrecht, the Netherlands, is the legal sponsor, and responsible for developing, implementing and managing the study in accordance with the protocol and all applicable laws and regulations. The study protocol has been approved by the medical ethics committees of the participating centres.

### Study population

Consecutive patients receiving an ICD or CRT-defibrillator (CRT-D) at one of the participating centres will be screened for study participation. Patients implanted with a first-time (primary or secondary prophylactic) ICD or CRT-D compatible with the LATITUDE® Patient Management system from Boston Scientific, with left ventricular ejection fraction ≤35 % and symptomatic heart failure (New York Heart Association functional class II or III) at the time of implantation, and providing written informed consent will be eligible to participate. Patients will be excluded if they are younger than 18 or older than 85 years of age, on the waiting list for heart transplantation, have a history of psychiatric illness other than affective/anxiety disorders, or are unable to complete the questionnaires due to cognitive impairments or insufficient knowledge of the language.

### Study procedure, randomisation and follow-up

When scheduled for device implantation, eligible patients receive a letter describing the purpose, design and possible benefits/risks of the study. Patients willing to participate are asked to sign an informed consent form. Of note, study refusal or withdrawal due to the patient having a strong preference for remote monitoring or in-clinic follow-up will be registered.

At discharge from hospital after implantation, included patients receive the baseline questionnaire and are asked to complete this 1 to 2 weeks after implantation (T_0_) to avoid measuring preoperative distress. When the completed T_0_ questionnaire is received at Tilburg University–which serves as core lab for the patient-reported outcomes–patients are randomised in a 1:1 fashion to either remote patient monitoring + in-clinic follow-up (RPM group) or in-clinic follow-up only (In-Clinic group) with the use of a blocked randomisation procedure. To ensure that the relative percentage of ICD and CRT-D patients is equal in both groups, we use separate randomisation procedures within these two subsets of patients.

**Fig. 1:**
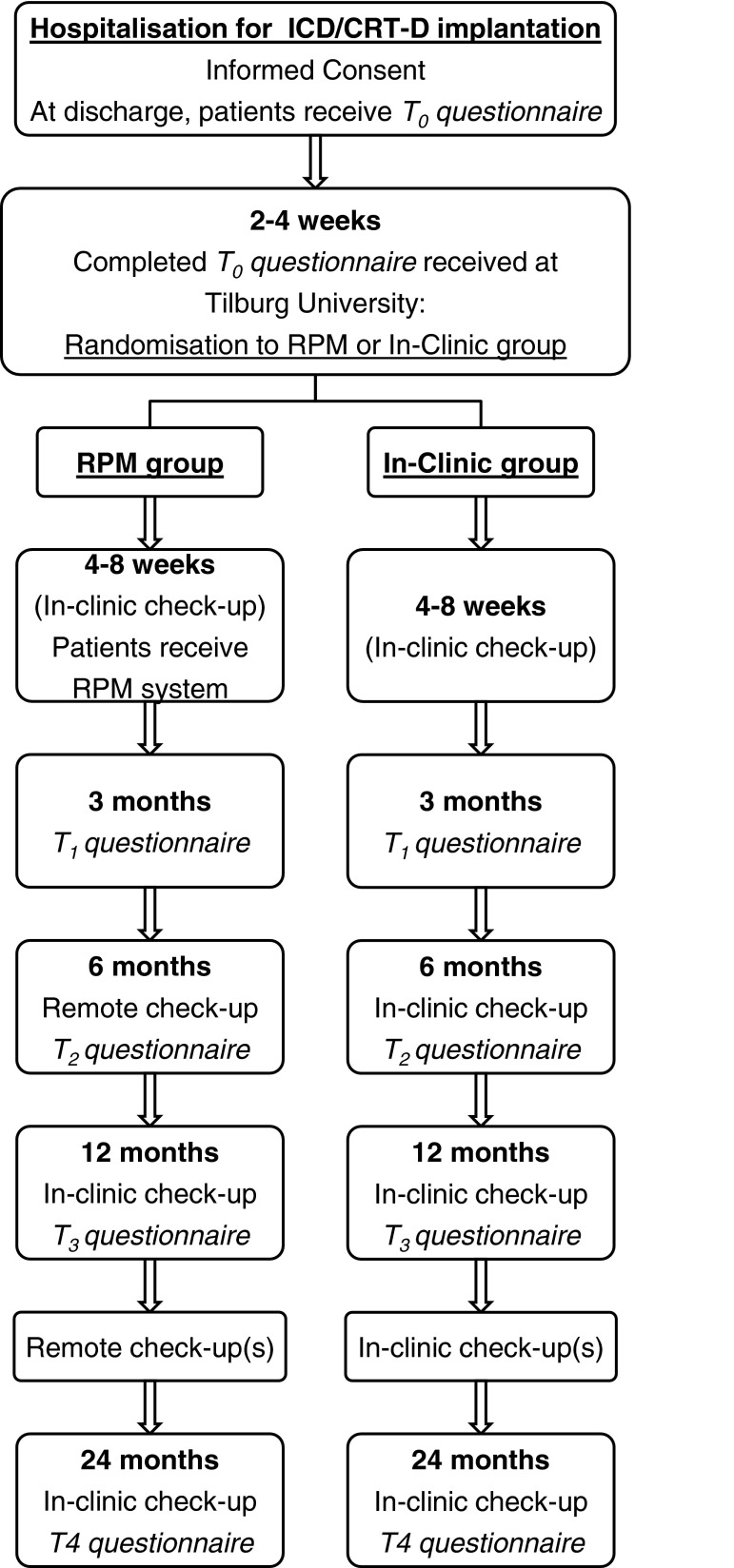
Schematic representation of the study design

Four to 8 weeks after implantation (preferably during the first in-clinic visit for assessment of wound healing etc.), patients in the RPM group will receive the remote monitoring system and be instructed how to install and use it. In accordance with the ACC/AHA/HRS guidelines, patients randomised to this group will visit the outpatient clinic again at 12 and 24 months after implantation [2]. The intermediate check-ups will be performed remotely. Patients randomised to the In-Clinic group will be followed up according to standard practice at the participating centres and visit the outpatient clinic (at least) every 3–6 months during the study. For patients in both groups, the follow-up assessments will take place at 3 (T_1_), 6 (T_2_), 12 (T_3_), and 24 (T_4_) months post-implantation. A schematic representation of the study design is shown in Fig. 1.

### Measures

Primary endpoints are patient-reported heart failure-specific health status (i.e., symptoms, functioning and quality of life), ICD acceptance and satisfaction with care. To assess these patient-reported outcomes and potentially associated psychological factors (i.e., Type D personality, psychological distress), patients in both groups will complete a number of questionnaires at the five assessment times (Table 1). All questionnaires are standardised and validated, except for the questionnaires used to assess patient satisfaction with remote monitoring, and their productivity and healthcare utilisation for cost-effectiveness. For these purposes, new questionnaires were developed.

**Table 1:** Patient-reported outcomes and psychological factors assessed in the study

Construct	Questionnaire	T_0_	T_1_	T_2_	T_3_	T_4_
Outcome measures						
Health status	Kansas City Cardiomyopathy Questionnaire (KCCQ)	x	x	x	x	x
Device acceptance	Florida Patient Acceptance Survey (FPAS)	x	x	x	x	x
Satisfaction with care	Visual analogue scale (0–100)	x	x	x	x	x
Satisfaction with remote monitoring^a^	Purpose-designed questionnaire		x	x	x	x
Cost-effectiveness: QALY	Euroqol 5D (EQ-5D) + VAS scale	x	x	x	x	x
Cost-effectiveness: productivity and health care utilisation	Purpose-designed questionnaire		x	x	x	x
Psychological variables						
ICD concerns	ICD Patient Concerns Questionnaire (ICDC)	x		x	x	x
ICD expectations^b^	Expectations regarding ICD	x				
Illness perception	Illness Perceptions Questionnaire (IPQ)	x			x	
Anxiety symptoms	Generalised Anxiety Disorder (GAD-7)	x			x	x
Depressive symptoms	Patient Health Questionnaire (PHQ-9)	x			x	x
Type D personality	Type D Scale (DS14)	x				
Self-care behaviour	European Heart Failure Self Care Behaviour Scale (EHFScBS)	x			x	x

T_0_ = Baseline; T_1_ = 3 months; T_2_ = 6 months; T_3_ = 12 months; T_4_ = 24 months

*ICD* implantable cardioverter defibrillator; *QALY* quality-adjusted life year; *VAS* visual analogue scale

^a^Patients in RPM group only

^b^Dutch patients only

Information on clinical characteristics and outcomes, including disease status, comorbidities, use of cardiac and psychotropic medication, ventricular arrhythmias, appropriate and inappropriate ICD therapy, in-clinic visits, hospital admissions, and mortality will be obtained from patients’ medical records or through device interrogation and gathered via electronic case report forms (Research Online) at baseline (T_0_), 6 (T_2_), 12 (T_3_) and 24 (T_4_) months after implantation.

### Remote patient monitoring system

Four to 8 weeks after implantation, patients randomised to the RPM group will receive the LATITUDE® Patient Management system, including weight scale and blood pressure cuffs, and will be instructed by an experienced ICD technician/nurse at the centre how to install and use it.

The LATITUDE® Patient Management system consists of a wireless communicator, a piece of equipment in the patients’ home, which is intended to remotely communicate with a compatible CIED from Boston Scientific and transfer data to a central database. The information gathered by the communicator is accessible to the patient’s healthcare team via the secured LATITUDE® website. Through the website, the clinician sets two automatic device interrogation schedules: (1) Remote device follow-ups: information similar to that of an in-clinic device interrogation is collected, including a real-time electrocardiogram (ECG), tests of battery status, lead impedances, and sensing amplitude; (2) Remote monitoring: during and in-between follow-ups, the clinic is notified when a predefined alert is detected. The communicator provides two levels of alerts: (a) red alerts indicate urgent conditions such as low life battery, low or high shock lead impedance, and possible device malfunction; (b) yellow alerts can be selected by the clinician and include various indications such as explant indicator reached, arrhythmias, and weight change. The clinician can also control patient-initiated, off-cycle data transmission if the patient needs to send data other than during routine follow-up or monitoring, for example in case of an ICD shock.

The LATITUDE® Heart Failure Management system includes tools to monitor the patient’s disease status. Patients can measure their weight and blood pressure by using the external weight scale and blood pressure monitor. These data are transmitted wirelessly to the LATITUDE® communicator.

Beside actions and measurements within the scope of this study, patients are treated according to the standard practice of their ICD centre.

### Sample size calculation and statistical analyses

The number of patients required to provide sufficient power to test the first objective of the proposed study was derived from a power analysis based on a small expected between-group effect size of .20, as measured with Cohen’s *d*. With alpha = 0.05 and power = 0.85 (two-sided test), 900 patients are needed (i.e., 450 in each group). The data will be analysed according to the intention-to-treat principle, with the inclusion of all randomised patients in the statistical analysis regardless of whether they completed the study.

Univariable and multivariable linear and logistic regression analyses will be performed to examine the group effects on health status, device acceptance and satisfaction with care at several points in time. Analysis of (co)variance with repeated measures will be performed to assess between-group differences in changes in these patient-reported outcomes over 2 years.

Patients’ answers on the ‘Satisfaction with remote monitoring’ questionnaire will be used to classify them into subgroups of patients that are/are not satisfied with remote monitoring. Chi-square tests and Student’s t-tests (or a non-parametric equivalent if necessary) will be performed to explore the demographic, clinical, and psychological characteristics of the patients in these subgroups. Also, multivariable logistic and linear regression analyses will be performed to examine which factors are independently associated with satisfaction with remote monitoring as a dichotomous outcome (satisfied: yes/no) and as a continuous outcome (satisfaction visual analogue scale 0–10), respectively.

To determine the cost-effectiveness, both short-term and long-term country-specific cost-effectiveness analyses will be performed. The short-term analysis has a 2-year time horizon, while the long-term analysis will have a lifetime horizon. Data used for the short-term analysis will be based on the data captured during the trial, while data used for the long-term analysis will consist of a combination of trial data and data from other sources. These data will be combined using a disease progression and treatment model and will be analysed using probabilistic analyses, sensitivity analyses, and scenario analyses. The primary cost-effectiveness outcome will be the incremental cost per quality-adjusted life year (QALY) gained.

## Discussion

Large-scale trials have shown that remote patient monitoring is a safe, effective and timely alternative for conventional in-clinic follow-up of ICD/CRT-D patients [6]. However, these trials have fallen short in their patient-centredness and have left an important question unanswered: namely, what do patients think about remote monitoring [12]? To date, evidence on the patient perspective on remote monitoring is scarce and inconsistent. As emphasised in a recent scientific statement from the American Heart Association, the assessment of patient-reported outcomes such as patient-reported health status is essential to enhance the patient-centredness of care and better characterise the impact of healthcare delivery on patient health [26]. No study has examined whether there is a subset of patients who might not benefit from remote monitoring in terms of patient-reported outcomes and would be more satisfied with standard clinical follow-up visits, and whether they are distinguishable based on their demographic, clinical, or psychological profile. The REMOTE-CIED study is an international, prospective randomised controlled study specifically designed to provide more insight into the patient perspective on and cost-effectiveness of remote monitoring compared with in-clinic follow-up in ICD/CRT-D patients. This study will be able to show how patients feel about remote monitoring and determine whether there are subgroups of patients that might be better served by receiving in-clinic follow-up visits. The study aims to include 900 consecutive patients from five European countries, who will be followed-up for 2 years post-implantation.

## Conclusion

The international REMOTE-CIED study is the first prospective randomised controlled study that primarily aims to assess the effect of remote patient monitoring on patient-reported outcomes in ICD patients, which could help to support patient-centred care in the future. The study is currently recruiting patients and results are expected in 2017.
